# Two Birds With One Stone: RNA Virus Strategies to Manipulate G3BP1 and Other Stress Granule Components

**DOI:** 10.1002/wrna.70005

**Published:** 2025-04-01

**Authors:** Moh Egy Rahman Firdaus, Eliana Dukhno, Rupali Kapoor, Piotr Gerlach

**Affiliations:** ^1^ IMol Polish Academy of Sciences Warsaw Poland; ^2^ ReMedy International Research Agenda Unit IMol Polish Academy of Sciences Warsaw Poland

**Keywords:** G3BP1, RNA viruses, stress granules, viral proteins

## Abstract

Stress granules (SGs) are membrane‐less organelles forming in the cytoplasm in response to various types of stress, including viral infection. SGs and SG‐associated proteins can play either a proviral role, by facilitating viral replication, or an antiviral role, by limiting the translation capacity, sequestering viral RNA, or contributing to the innate immune response of the cell. Consequently, viruses frequently target stress granules while counteracting cellular translation shut‐off and the antiviral response. One strategy is to sequester SG components, not only to impair their assembly but also to repurpose and incorporate them into viral replication sites. G3BP1 is a key SG protein, driving its nucleation through protein–protein and protein–RNA interactions. Many cellular proteins, including other SG components, interact with G3BP1 via their ΦxFG motifs. Notably, SARS‐CoV N proteins and alphaviral nsP3 proteins contain similar motifs, allowing them to compete for G3BP1. Several SG proteins have been shown to interact with the flaviviral capsid protein, which is primarily responsible for anchoring the viral genome inside the virion. There are also numerous examples of structured elements within coronaviral and flaviviral RNAs recruiting or sponging SG proteins. Despite these insights, the structural and biochemical details of SG‐virus interactions remain largely unexplored and are known only for a handful of cases. Exploring their molecular relevance for infection and discovering new examples of direct SG‐virus contacts is highly important, as advances in this area will open new possibilities for the design of targeted therapies and potentially broad‐spectrum antivirals.

## Graphical abstract

Stress granules emerging during viral infection can play both pro‐ and antiviral roles. RNA viruses use various strategies to antagonize their formation, including the degradation, inhibition, or sequestration of G3BP1, a key stress granule protein. However, biochemical and structural insights into these mechanisms have been reported for only a handful of cases. Further advancements in this area are crucial, as they may pave the way for the development of targeted therapies and antiviral drugs

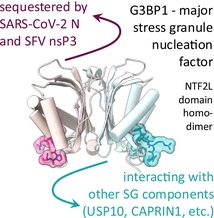

## 1 Introduction

Upon exposure to stress, including the one caused by viral infections, cells tend to reorganize their translation landscape. The balance between translating and non‐translating pools of mRNA gets shifted towards translation inhibition, at the same time redirecting cellular resources and energy towards the synthesis of the innate immune‐related defense weaponry. Translation inhibition is most efficiently achieved by arresting its rate‐limiting initiation step – either by proteolytic degradation or PTM alteration of the 5′ cap‐binding eIF4F complex, or by the eIF2α phosphorylation, resulting in depletion of the available ternary complexes (Anderson and Kedersha 2002). While actively translated mRNAs are associated with polysomes, the non‐translated, polysome‐free mRNA are either targeted to processing bodies (PB), where they can be turned over by decapping enzymes and exonucleases, or they populate stress granules (SG) – dynamic, membrane‐less foci considered in some reports as an intermediate in the mRNA transition between polysomes and PBs (Balagopal and Parker 2009; Buchan and Parker 2009; Chantarachot and Bailey‐Serres 2018; Kedersha et al. 2005; Parker and Sheth 2007). While PBs are constitutive entities in most cell types, SGs form upon stress or overexpression of their components. By accumulating translation factors, particularly stalled translation initiation complexes and non‐translating mRNA, SGs are supposed to buffer cellular translation capacity, at the same time contributing to the cytoprotective mode of the metabolism (Hilgers et al. 2006). Alternatively, increased concentration of mRNAs and proteins might in fact enhance the assembly of translation initiation complexes and facilitate the preferential translation of specific mRNAs (Spriggs et al. 2008), as visualized by single‐molecule imaging (Mateju et al. 2020).

Considering that ribosome‐free mRNAs and the surrounding RNA‐binding proteins are likely to condense into RNA granules, SGs may even be regarded as byproducts and not regulators of the translation rearrangement in stress (Mateju and Chao 2022). Disassembly of typically transient SGs is mediated by autophagy, chaperones, ATP‐dependent remodelers of RNPs, RNA helicases, and post‐translational modifications of RNA‐binding SG proteins (Hofmann et al. 2021; Wheeler et al. 2016). Dysregulation of SG formation or their timely disassembly and clearance can cause various pathologies, like neuro‐ or muscular degenerative diseases (Dobra et al. 2018; Fang et al. 2024; Wolozin and Ivanov 2019), and aging‐related diseases (Cao et al. 2020). Upregulated SGs contribute as well to the progression of various tumors (Li et al. 2023; Zhou et al. 2023).

During viral infections, SGs and SG‐associated proteins were shown to play both antiviral and proviral roles (Brownsword and Locker 2023; Guan et al. 2023; Jayabalan et al. 2023; Poblete‐Durán et al. 2016; Zhang, Sharma, et al. 2019). Beyond their role in limiting the pool of virus‐accessible translation machinery, SGs were also proposed to serve as innate immune response hubs. By recruiting and condensing antiviral signaling proteins like RIG‐I, MDA5, PKR, OAS, and RNaseL, SGs enhance their interactions with sequestered viral RNAs and stimulate their activation, thus contributing to the amplification of the innate immune signaling (Eiermann et al. 2020; McCormick and Khaperskyy 2017; Onomoto et al. 2012, 2014). Particularly, G3BP1, G3BP2, and CAPRIN1 were proposed to act as positive regulators of the antiviral IFN response, contributing to the interferon‐stimulated genes (ISG) mRNAs translation (Bidet et al. 2014; Reineke and Lloyd 2015). Conversely, a mere condensation and sequestration of viral mRNAs into SGs may not only inhibit their translation but can also buffer the amount of recognizable foreign RNAs, preventing an over‐activation of the antiviral signaling, which, beyond a certain threshold, could lead to cell collapse and death (Paget et al. 2023).

Apart from SGs, dsRNA‐induced foci (dRIF), RNase L‐dependent bodies (RLBs), or paracrine SG‐like granules formed in the bystander cells are also observed to form during viral infections (Burke et al. 2020; Corbet et al. 2022; Iadevaia et al. 2022; Watkins and Burke 2024).

Since SGs contribute to translation reshuffling and may facilitate the activation of innate immune signaling, many viruses use a broad range of approaches to block or neutralize them. Viral strategies to destabilize SG formation by sequestering or even repurposing SG components have mostly been studied by co‐immunofluorescence and co‐immunopurification. These approaches cannot unambiguously confirm direct interactions between host and virus proteins and do not provide mechanistic insight. The intention of this mini‐review is to comment on the cross‐talk between RNA viruses and SGs, discussing contacts between viral and SG factors and focusing in detail on the biophysically and structurally determined interactions. Until now, unambiguous protein–protein interactions have only been described for coronaviral nucleoprotein and alphaviral nsP3 protein, both binding and sequestering G3BP1. Specific interactions between viral RNAs and SG proteins, particularly in the case of flaviviral genomic UTRs, are well documented as well, but detailed structural insight is lacking. In many cases, SG proteins get recruited into viral replication centers, but the molecular mechanisms of their contribution remain vague. Further exploration of direct interactions between SG components and viral factors is needed, as they might prove therapeutically relevant.

## 2 Stress Granules

### 2.1 Assembly, Composition and Dynamics

Archetypical SG assembly depends on phosphorylation of the serine 51 residue of eIF2α, mediated by four integrated stress response (ISR) serine/threonine kinases, stimulated by: amino acid deprivation (GCN2 kinase); oxidative stress, heat shock, and osmotic stress (HRI kinase); ER stress in response to the accumulation of misfolded proteins (PERK kinase); or viral infection and the presence of double‐stranded viral RNAs (PKR kinase) (Wek et al. 2006). Phosphorylation of the α subunit of the eIF2 trimer prevents the exchange of GDP for GTP within its γ subunit by the decameric nucleotide exchange factor eIF2B (Adomavicius et al. 2019; Gordiyenko et al. 2019; Kashiwagi et al. 2019). This consequently leads to lower availability of the eIF2 ternary complex (eIF2‐GTP‐tRNAi), indispensable during 5′ cap‐dependent translation initiation. Alternatively, SG assembly can be triggered by misregulation of the eIF4F complex, resulting from the eIF4E 5′ cap‐binding factor sequestration by the dephosphorylated 4EBP (Peter et al. 2015), inhibition of the eIF4A helicase, or cleavage of the eIF4G by viral proteases.

SGs assemble through RNA–RNA, RNA–protein, and protein–protein interactions (Matheny et al. 2021; Mittag and Parker 2018; Tauber et al. 2020; Van Treeck and Parker 2018), often involving low‐complexity domains or intrinsically disordered regions (IDRs), providing sites for multivalent weak interactions and promoting liquid–liquid phase separation (LLPS) (Lee et al. 2022; Molliex et al. 2015). Around 10%–15% of the non‐translating cytoplasmic long mRNAs, released from the polysomes upon translation shutoff, self‐assemble via promiscuous or specific RNA–RNA interactions (Khong et al. 2017), providing scaffolds attracting SG‐associated RNA‐binding proteins, altogether leading to the formation of entangled, higher‐order assemblies driving SG formation (Van Treeck and Parker 2018). It starts with the nucleation of dense and stable cores, which then become surrounded by liquid‐like and more dynamic shells (Cirillo et al. 2020; Jain et al. 2016; Protter and Parker 2016). SGs then grow by expansion and fusion of the shells (Ohshima et al. 2015; Wheeler et al. 2016), assisted and regulated by microtubules (Chernov et al. 2009; Loschi et al. 2009). Electron and super‐resolution microscopy visualized the core‐shell SG structure, with ~180 nm cores, ~30× denser than the cytoplasm, surrounded by less concentrated shells (Jain et al. 2016; Niewidok et al. 2018; Souquere et al. 2009; Treeck and Parker 2019). According to fluorescence recovery after photobleaching (FRAP) measurements, SG proteins dynamically exchange with the surrounding cytoplasm, with rates ranging from seconds to minutes (Buchan and Parker 2009), although proteins located in SG cores show prolonged residence.

### 2.2 Stress Granule Proteins

Mass spectroscopy analysis of the purified SG cores (Jain et al. 2016) or proximity‐based proteomics (Markmiller et al. 2018; Youn et al. 2018) have expanded the number of mammalian SG proteins from initially determined 75 (Buchan and Parker 2009) to approximately 300, yielding novel, often stress‐specific, protein–protein interactions. Major SG protein components are G3BP1/2, ATXN2/2L, UBAP2L, TIA1/TIAR, CAPRIN1, USP10, TRIM25, TRIM56, DDX3X, FMR1, FXR1, PRRC2C, CSDE1, TAF15, NUFIP2, HDAC6, PABPC1, 40S small ribosomal subunit, and EIF3 factor components (Gilks et al. 2004; Kedersha et al. 2016, 1999; Kwon et al. 2007; Markmiller et al. 2018; Sanders et al. 2020; Solomon et al. 2007; Yang et al. 2020; Youn et al. 2018). IDRs of the SG‐associated proteins frequently contain short linear motifs (SLiMs), specifically interacting with well‐folded domains of their interacting partners. When interacting SG proteins contain, in addition, RNA‐binding domains (RBD), it increases the collective number of RNA contacts, thus lowering the RNA concentration threshold for the RNA–protein LLPS, cooperatively and synergistically influencing the assembly and stability of SGs (Sanders et al. 2020; Yang et al. 2020). Interestingly, many SG proteins, including G3BP1, were shown in fact to partake in pre‐existing networks that can prime rapid SG assembly (Markmiller et al. 2018; Youn et al. 2018). In addition, ATP‐dependent remodeling complexes like protein chaperones or RNA helicases like EIF4A are also involved in SG formation, fusion, and components exchange events (Jain et al. 2016).

### 2.3 Stress Granule RNAs

In contrast to proteins, most mRNAs sequestered in SGs have much slower exchange rates, and they are usually released upon SG disassembly (Zhang et al. 2011). Approximately 80% of the RNAs accumulated in mammalian stress granules are mRNAs, with long UTRs and coding sequences (Khong et al. 2017), frequently rich in ARE elements (Namkoong et al. 2018). Single‐molecule imaging confirmed that long and non‐translating mRNAs have longer resident times in SGs (Moon et al. 2019) and showed that most of them are recruited within the first 30 min of stress induction (Wilbertz et al. 2019). On the other hand, given that SG‐less cells still efficiently repress global translation (Bley et al. 2015; Kedersha et al. 2016) and that SGs accumulate only a fraction of the cytoplasmic mRNA (Khong et al. 2017), their indispensability for translation shut‐off is questionable. In fact, since SG‐accumulated mRNAs are enriched for transcripts associated with cell survival and growth regulation, SG sequestration might as well play a protective, rather than a translation‐inhibitory role during stress (Namkoong et al. 2018).

### 2.4 G3BP1 — The Major SG‐Associated Protein Component

G3BP proteins are the central protein components of stress granules (Alam and Kennedy 2019). They derive their name from the original discovery identifying G3BP1 as a Ras‐GTPase‐activating protein SH3 domain‐binding protein (Parker et al. 1996), although that binding has later been questioned (Annibaldi et al. 2011). Over the last few years, G3BP1 emerged as a significant cancer marker protein and a drug target, overexpressed in various cancer tumors (Zhang, Wang, et al. 2019). It was also shown to tether the TSC complex to lysosomes, thus negatively regulating mTORC1 signaling (Prentzell et al. 2021), or to enhance cytosolic DNA binding by cGAS, priming it to form large complexes or condensates and to trigger the interferon response (Liu et al. 2019; Zhao et al. 2022). Additionally, cytoplasmically distributed G3BP1 was proposed as well to stabilize and upregulate short mRNAs (Laver et al. 2020), or to partake in UPF1‐mediated degradation of mRNAs with structured 3′ UTRs (Fischer et al. 2020).

G3BP1 is primarily known, though, as a central node governing SG assembly, both through protein–protein interactions and through RNA‐dependent LLPS. It is worth noting, though, that UBAP2L has been proposed as an alternative, G3BP1‐independent SG nucleator, and that UBAP2L cores facilitate subsequent G3BP1 core formation, required for SG maturation (Cirillo et al. 2020; Huang et al. 2020). Association between UBAP2L and G3BP1 was proposed to be mediated by snoRNAs (Asano‐Inami et al. 2023), and UBAP2L was additionally shown to act as a bridging factor between SGs and PBs (Riggs et al. 2024).

#### 2.4.1 G3BP1 — Domain Organization, RNA Binding, LLPS, and PTMs

**FIGURE 1:**
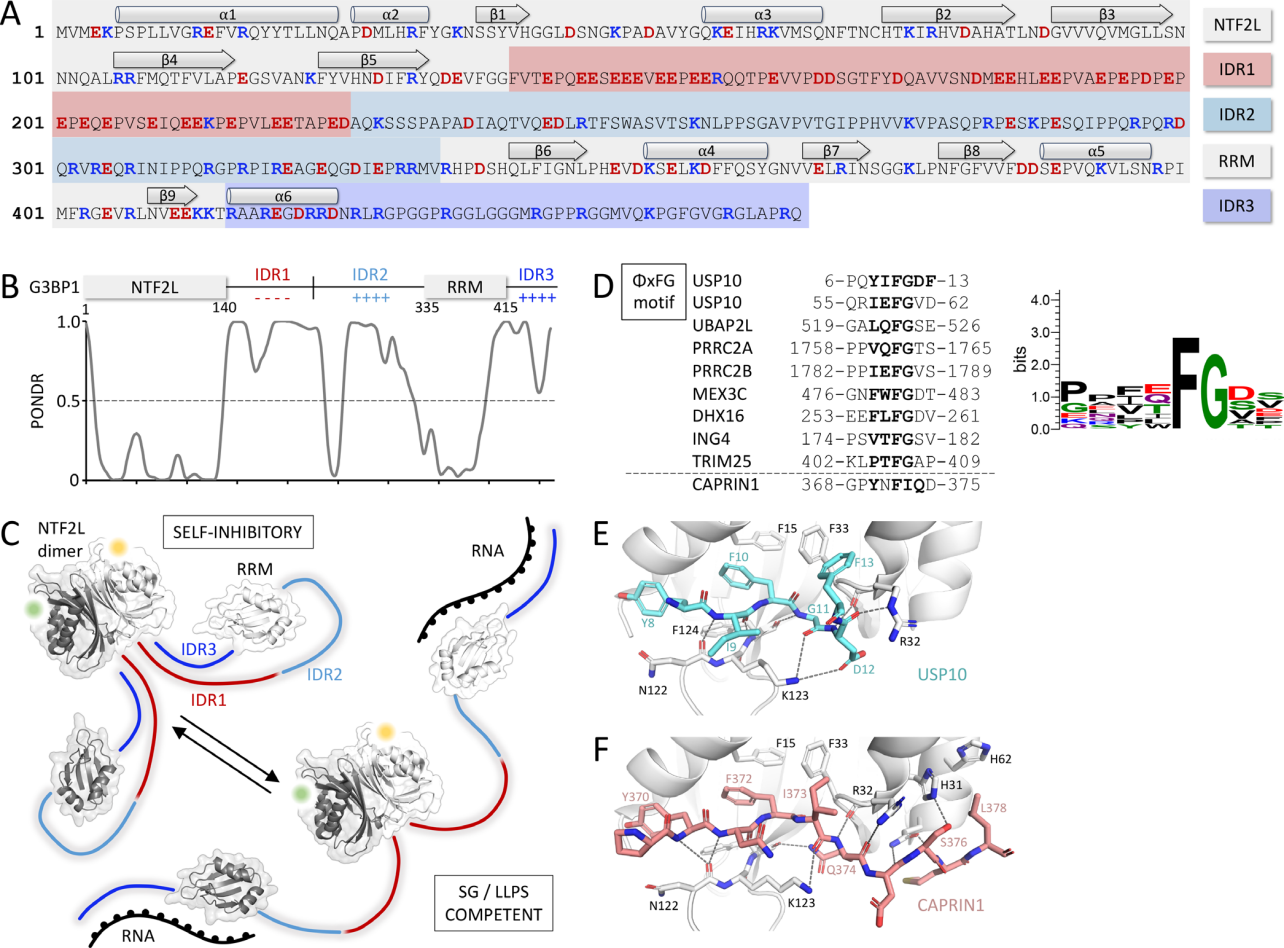
G3BP1 features and interactions with other stress granule components. (A) G3BP1 sequence with secondary structure elements and functional regions highlighted; (B) G3BP1 regions aligned with PONDR (predictor of naturally disordered regions) estimations; (C) Transition between self‐inhibitory and SG/LLPS‐competent G3BP1 states; Panels A, B, and C were adapted from Yang et al. 2020; (D) Sequence alignment and logo representation of the G3BP1 NTF2L‐interacting ΦxFG motif in some stress granule factors, adapted from Kruse et al. 2021; (E) USP10 peptide bound to the G3BP1 NTF2L domain (PDB: 7XHF, Song et al. 2022); (F) CAPRIN1 peptide bound to the G3BP1 NTF2L domain (PDB: 7XHG, Song et al. 2022).

There are three isoforms of G3BP in human cells: the G3BP1 and two splice variants of the G3BP2 – 2a and 2b (Kennedy et al. 2002). The main difference between these isoforms is the number of PxxP motifs in the central region of the protein. G3BP1 is a 466‐residue‐long, modular protein composed of the nuclear transport factor 2‐like (NTF2L) domain, followed by two IDRs (the first one being reach in glutamic acids (pI = 3.4)), the RNA recognition motif (RRM), and the third, positively charged and RG‐rich IDR (Figure 1A,B). Similarly to the NTF2 homodimer (Bayliss et al. 2002), the NTF2L domain of G3BP1 dimerizes through an extended β‐sheet‐to‐β‐sheet interaction (buried surface area of ~8000 Å^2^). IDRs of G3BP1 modulate its interaction with single‐stranded RNAs, impacting consequently its LLPS predisposition. The acidic IDR1 acts as the G3BP1 self‐inhibitory element, preventing the RG‐rich IDR3 from interacting with RNA. Above a certain threshold concentration, though, free RNA binds to the G3BP1 RRM and IDR3, outcompeting the acidic IDR1. This transits G3BP1 from a “closed” self‐inhibitory state into an “open” LLPS‐prone state (Guillén‐Boixet et al. 2020; Yang et al. 2020) (Figure 1C). Both dimerization of G3BP1 and its multivalency for RNA binding synergistically mediate SG formation and stability.

Post‐translational modifications of G3BP1 were shown to regulate its LLPS and SG‐formation capacities. Phosphorylation of S149, within the E‐rich region of the IDR1 was shown to enable fine‐tuning of the SG assembly and disassembly, by strengthening the IDR1–IDR3 self‐inhibitory interaction and making it more difficult to establish the G3BP1–RNA network (Yang et al. 2020). On the other hand, the G3BP1 S149 phosphorylation status was demonstrated not to change upon stress, arguing that it is not a simple on‐and‐off SG formation switch (Panas et al. 2019). Phosphorylation of Y40 was proposed to promote the NTF2L domain‐driven G3BP1 dimerization (Kim et al. 2022). In addition, methylation of arginines within the RG‐rich IDR3 masks positive charges of the RNA‐biding residues, negatively regulating the G3BP1–RNA interactions (Tsai et al. 2016). ADP ribosylation of G3BPs and other SG components was also suggested to regulate LLPS dynamics and promote SG formation (Jayabalan et al. 2021).

#### 2.4.2 G3BP1 — Interaction With the ΦxFG SLiM‐Containing Partners

Another aspect making G3BP1 a central and versatile player within the SG network is its propensity to interact with a wide range of other SG‐associated proteins. The G3BP1 NTF2L domain has a hydrophobic cleft, located opposite the dimerization surface (Figure 1C), that can recruit other SG proteins via their SLiMs with a consensus ΦxFG sequence (where Φ is a hydrophobic residue) (Figure 1D). So far, the interaction between the G3BP1 NTF2L domain and the SLiM of another SG protein has been structurally determined only for the ubiquitin‐specific protease 10 (USP10) and CAPRIN1 (Schulte et al. 2023; Song et al. 2022) (Figure 1E). In both cases, the key SLiM residue is a phenylalanine (F10 in USP10 and F372 in CAPRIN1), pointing towards an NTF2L hydrophobic pocket defined by phenylalanines F15, F33, and less crucial F124. The G3BP1 NTF2L F33W mutation was shown to abrogate the interaction with USP10 and CAPRIN1 (Kedersha et al. 2016). The ΦxFG glycine residue is also important, as it allows the peptide to turn, leading to an adequate positioning of the downstream residues (Panas et al. 2015). In USP10, these are D12, which flips away from the NTF2L groove, and F13, which recontacts the NTF2L F33 (Song et al. 2022). Instead of G‐D‐F residues, CAPRIN1 isoleucine I373 is positioned similarly to the USP10 F13 and also contacts the NTF2L F33. In addition, CAPRIN1 expands its binding, with both S376 and L378 reaching the NTF2L histidines H31 and H62 (Schulte et al. 2023; Song et al. 2022).

Since SLiMs from different SG proteins have different affinities to the G3BP1 NTF2L and different outcomes on the SG assembly, the NTF2L‐SLiM interaction is yet another mechanism regulating SG assembly and stability. While the interaction between G3BP1 NTF2L and CAPRIN1 or UBAP2L promotes SG assembly, the interaction with USP10 limits it (Kedersha et al. 2016; Markmiller et al. 2018; Panas et al. 2015; Youn et al. 2018). This is because USP10, which lacks an RBD, acts as an RNA valence cap and decreases the RNA‐binding capacity of the SG protein network (Sanders et al. 2020). Interestingly, the G3BP1–USP10 interaction is mutually inhibitory, as it inhibits the deubiquitinase activity of USP10 (Soncini et al. 2001).

## 3 RNA Virus Strategies Targeting Stress Granules and G3BP1

As introduced above, SGs and SG‐associated proteins may play both antiviral and proviral roles. Apart from entrapping viral RNAs and sponging translation machinery that viruses rely on, SGs were proposed as well to facilitate the immune response. For these reasons, many viruses use various strategies to block or neutralize them.

Given that G3BP1 is a central protein component of stress granules, a growing number of data shows that it is one of the key host proteins impacting viral life cycles, both positively and negatively (Jayabalan et al. 2023). In some cases G3BP1 inhibits viral replication, for instance by directly assisting RIG‐I in the viral RNA recognition and signaling, and by maintaining RIG‐I levels (Kim et al. 2019; Yang et al. 2019).

Some RNA viruses, like picornaviruses, are equipped with proteases targeting several host factors involved in translation regulation, including G3BP1. Others, like noroviruses or flaviviruses, sequester G3BP1 from disassembled SGs and recruit it to replication sites where it starts contributing to viral propagation (Bonenfant et al. 2019; Hosmillo et al. 2019). This proves that some viruses may target SGs not only to disassemble them, preventing their anti‐viral role, but also to exploit SG components, abusing their features and capacities. In the case of alpha‐ and coronaviruses, the binding of the viral protein SLiMs to the G3BP1 NTF2L domain has been characterized biochemically and structurally (Biswal et al. 2022; Schulte et al. 2016). The inhibitory impact of these interactions on SG formation has inspired the design of peptide mimetics, which could potentially be used in various cancers and neurodegenerative diseases with upregulated SGs (Freibaum et al. 2024).

### 3.1 Alphaviruses

#### 3.1.1 Stress Granules Disassembly and G3BP1 Recruitment Into Viral Replication Sites

Upon alphaviral infection, the host translation shuts off due to the PKR‐mediated phosphorylation of the eIF2α. Alphaviruses seem to tolerate those circumstances since the G:C‐rich downstream loop promoter (DLP) feature, located within their mRNA protein‐coding region 5′ end, allows for eIF2α‐independent translation initiation (Carrasco et al. 2018). As shown for the Semliki Forest virus (SFV), eIF2α(P)‐triggered SG formation starts early in infection, but SGs disassemble within hours, starting with those located near the viral RNA replication sites (McInerney et al. 2005). Interestingly, despite SG disappearance, host translation remains low as the eIF2α(P) persists. Disassembly of SGs during alphaviral infection is mediated both by the interaction between viral nonstructural protein 3 (nsP3) and G3BP1 (Panas et al. 2012) and by mere inhibition of cellular transcription and translation (Frolova et al. 2023). Old World alphavirus nsP3 proteins sequester G3BP1 from SGs and relocate it into nsP3‐mediated foci, forming near the plasma membrane‐attached viral replicative spherules, also known as viral replication complexes (vRC) (Fros et al. 2012; Kril et al. 2021; Tan et al. 2022). In fact, G3BP1 plays an important proviral role within these structures, as Chikungunya virus cannot replicate its genome in the G3BP1‐depleted cells and SFV replication is substantially reduced (Scholte et al. 2015). The nsP3–G3BP1 matrix could form a protective layer for the newly synthesized viral RNA, shielding it from RNA degradation machinery or cytosolic dsRNA sensors (Kim et al. 2016). Importantly, host translation machinery is brought into the nsP3 foci near alphaviral vRCs – while G3BP1 NTF2L domain binds nsP3, its RG‐rich IDR3 associates with the 40S ribosomal subunit, suggesting that nsP3 uses G3BP1 to recruit the translation initiation machinery into vRCs, ensuring efficient translation of the viral genomic mRNAs (Götte et al. 2019). In contrast to Old World alphaviruses, New World alphavirus nsP3 proteins were shown to interact with proteins from the fragile X syndrome (FXR) family instead of G3BPs (Kim et al. 2016). Finally, rubella virus, originally assigned alongside alphaviruses within the *Togaviridae* family, induces formation of the G3BP1 foci. They are, however, proteomically distinct from SGs, as they do not contain other SG components like PABPC1 or TIA1 and are resistant to the cycloheximide treatment (Matthews and Frey 2012).

#### 3.1.2 Details of the Alphavirus nsP3–G3BP1 Interaction

**FIGURE 2:**
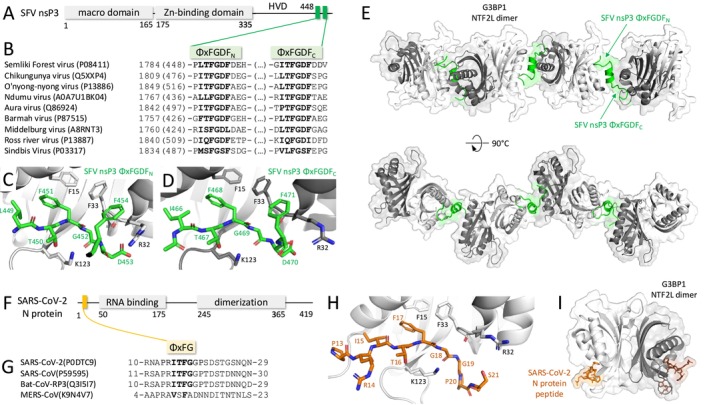
Viral protein interacting with G3BP1. (A) Domain organization of the Semliki Forest alphavirus nsP3 protein; (B) Alignment of selected alphaviral nsP3 proteins, highlighting conservation of the two ΦxFGDF motifs; (C) and (D) SFV nsP3 ΦxFGDF motifs biding to the G3BP1 NTF2L domain (PDB: 5FW5, Schulte et al. 2016); (E) Possible higher oligomer organization with a single SFV nsP3 protein binding two G3BP1 NTF2L homodimers; (F) SARS‐CoV‐2 N protein domain organization; (G) Alignment of selected coronaviral N proteins, highlighting conservation of the two ΦxFG motifs; (H) SARS‐CoV‐2 N protein ΦxFG motif biding to the G3BP1 NTF2L domain (PDB: 7SUO, Biswal et al. 2022); (I) Overview of two SARS‐CoV‐2 N protein ΦxFG motifs binding to a single G3BP1 NTF2L homodimer.

Due to its SG‐disrupting role, the nsP3 protein of the Old World alphaviruses, like SFV, Sindbis virus, or Chikungunya virus, is considered a host defense modulator. It is composed of an N‐terminal macrodomain (MD) with ADP‐ribose binding and hydrolytic capacities, followed by an alphavirus‐specific zinc‐binding domain (Shin et al. 2012), and a C‐terminal largely disordered hypervariable domain (HVD) (Figure 2A). The nsP3 N‐terminal MD was proposed to catalyze a decrease in G3BP1 ADP‐ribosylation, thus contributing to the SG disassembly (Jayabalan et al. 2021). The nsP3 HDV contains two conserved USP10‐like ΦxFGDF SLiM motifs and several other motifs mediating interactions with host proteins (Figure 2B). The amino acid sequence between the 1st motif (ΦxFGDF_N_, residues 449–454 in SFV) and the 2nd motif (ΦxFGDF_C_, residues 466–471 in SFV) folds into an α‐helix and is too short to span two SLiM‐interacting grooves within the G3BP1 NTF2L homodimer. Instead, the crystal structure of the G3BP1 NTF2L with the SFV nsP3 peptide (1–25) revealed that ΦxFGDF SLiMs from a single nsP3 engage two NTF2L homodimers (Figure 2C,D), (Table 1), allowing for the formation of an extended assembly of multiple G3BP1 dimers inter‐connected with the alphaviral nsP3 proteins in the form of (2 × G3BP1–nsP3)n oligomeric fiber (Schulte et al. 2016) (Figure 2E).

**TABLE 1:** Available structures of the G3BP NTF2L homodimer in complex with the interacting peptide.

G3BP construct	Interactor	PDB	Ref
G3BP1 (1–139)	—	3Q90	SGC 2011
G3BP1 (1–139)	—	4FCJ	Vognsen et al. 2013
G3BP1 (1–139)	Nucleoporin repeat peptide DS**GFSF**GSK	4FCM	
G3BP1 (11–139)	—	4IIA	
G3BP2 (1–139)	SFV nsP3 (449–456) peptide **LTFGDF**DE	5DRV	Kristensen 2015
G3BP1 (1–139)	SFV nsP3 (449–473) **LTFGDF**DEHEVDALASG**ITFGDF**DD	5FW5	Schulte et al. 2016
G3BP1 (1–139)	CAPRIN1 (356–386) RQRVQDLMAQMQGP**YNFI**QDSMLDFENQTLD	6TA7	Schulte et al. 2023
G3BP1 (1–139)	—	7S17	Sheehan et al. 2022
G3BP1 (1–139)	SARS‐CoV‐2 Nprotein (1–25) MSDNGPQNQRNAPR**ITFG**GPSDSTG	7SUO	Biswal et al. 2022
G3BP1 (1–139)	USP10 (1–40 but only 6–21 visible) PQ**YIFGDF**SPDEFNQF	7XHF	Song et al. 2022
G3BP1 (1–139)	CAPRIN1 (347–386 but only 369–378 visible) P**YNFI**QDSML	7XHG	
G3BP1 (1–139)	SARS‐CoV‐2 Alpha Nprotein (1–25, D3L) MSLNGPQNQRNAPR**ITFG**GPSDSTG	8TH1	Yang et al. 2024
G3BP1 (1–139)	SARS‐CoV‐2 Omicron Nprotein (1–25, P13L) MSDNGPQNQRNALR**ITFG**GPSDSTG	8TH5	
G3BP1 (1–139)	USP10 (1–24, M1G) GALHSPQ**YIFGDF**SPDEFNQFFVT	8TH6	
G3BP1 (1–139)	CAPRIN1 (360–381) QDLMAQMQGP**YNFI**QDSMLDFE	8TH7	
G3BP1 (1–139)	Peptide mimetic G3Ia	8V1L	Freibaum et al. 2024

Both SFV nsP3 motifs bind within the hydrophobic grooves of the bridged NTF2L domains in the exact same fashion, conformationally similar to the USP10. The first phenylalanines of both motifs (F451 and F468) locate within the NTF2L domain F15/F33 hydrophobic pocket, while the NTF2L lysine K123 locks the nsP3 motifs from the other side (Schulte et al. 2016). The α‐helix linker also contributes to the binding of the bridged NTF2L domains, with its charged and hydrophobic residues. Microscale thermophoresis (MST) and immunoprecipitations from transfected or infected cells revealed altogether that SFV nsP3 motifs bind NTF2L domains hierarchically and sequentially – the ΦxFGDF_N_ with sub‐μmolar and the ΦxFGDF_C_ with low μmolar affinity (Table 2), (Schulte et al. 2016). While mutation of the F451 in the ΦxFGDF_N_ motif fully abolishes recruitment of the G3BP1 into nsP3‐positive viral RNA replication complexes, mutation of the F468 in the ΦxFGDF_C_ only weakens it (Schulte et al. 2016). However, the interaction of both motifs with G3BP1 is necessary for efficient replication of SFV, CHIKV (Wang and Merits 2022), and possibly other Old World alphaviruses, as supported by their evolutionary conservation (Nowee et al. 2021).

**TABLE 2:** Overview of biophysical measurements of the affinity between the G3BP NTF2L and interacting peptides.

G3BP construct	Interactor	Kd (μM)	Method	Protein buffer	Ref
G3BP1 NTF2L WT	Nucleoporin repeat peptide GQSPG**FG**QGGSV	n.d.	ITC	100 mM Hepes pH 8.0, 100 mM NaCl	Vognsen et al. 2013
G3BP2 NTF2L WT	Nucleoporin repeat peptide GQSPG**FG**QGGSV	n.d.			
G3BP1 NTF2L WT	Nucleoporin repeat peptide DSG**GLFG**SK	n.d.			
G3BP2 NTF2L WT	Nucleoporin repeat peptide DSG**GLFG**SK	n.d.			
G3BP1 NTF2L WT	Nucleoporin repeat peptide DSG**FSFG**SK	115			
G3BP2 NTF2L WT	Nucleoporin repeat peptide DSG**FSFG**SK	114			
G3BP1 NTF2L F15A	Nucleoporin repeat peptide DSG**FSFG**SK	670			
G3BP1 NTF2L WT	SFV nsP3 WT (449–473) **LTFGDF**DEHEVDALASG**ITFGDF**DD	6.7	ITC	25 mM Hepes pH 7.5, 150 mM NaCl, 10 mM MgCl_2_, 10% Glycerol	Panas et al. 2015
G3BP1 NTF2L WT	SFV nsP3 MUT (449–473) **LTAGDA** DEHEVDALASG**ITAGDA** DD	n.d.			
G3BP1 NTF2L WT	SFV nsP3 WT (449–473) **LTFGDF**DEHEVDALASG**ITFGDF**DD	0.15/4.1	MST	20 mM Hepes pH 7.5, 300 mM NaCl, 5 mM MgCl_2_, 0.05% Tween‐20, 10% Glycerol, 2 mM DTT, 1 mg/mL BSA	Schulte et al. 2016
G3BP1 NTF2L WT	SFV nsP3 F3A_C_ (449–473) **LTFGDF**DEHEVDALASG**ITAGDF**DD	0.4/23			
G3BP1 NTF2L WT	SFV nsP3 F3A_N_ (449–473) **LTAGDF**DEHEVDALASG**ITFGDF**DD	5.8			
G3BP1 NTF2L WT	SFV nsP3 F3A_NC_ (449–473) **LTAGDF**DEHEVDALASG**ITAGDF**DD	17			
G3BP1 NTF2L WT	SARS‐CoV‐2 N WT (11–24) NAPR**ITFG**GPSDST	10	ITC	100 mM Tris pH7.5, 300 mM NaCl, 5% Glycerol	Huang et al. 2021
G3BP1 NTF2L WT	SARS‐CoV‐2 N I15A (11–24) NAPR **ATFG**GPSDST	70			
G3BP1 NTF2L WT	SARS‐CoV‐2 N F17A (11–24) NAPR**ITAG**GPSDST	70			
G3BP1 NTF2L WT	SARS‐CoV‐2 N 4xA (11–24) NAPR **AAAA** GPSDST	n.d.			
G3BP1 FL WT	SARS‐CoV‐2 N WT (FL)	1.0	ITC	25 mM Hepes pH 7.5, 300 mM NaCl	Biswal et al. 2022
G3BP1 FL WT	SARS‐CoV‐2 N WT (1–25) MSDNGPQNQRNAPR**ITFG**GPSDSTG	7.9			
G3BP1 FL WT	SARS‐CoV‐2 N WT (26–419)	n.d.			
G3BP1 FL WT	SARS‐CoV‐2 N WT (1–175)	10.9			
G3BP1 FL WT	SARS‐CoV‐2 N WT (1–364)	1.9			
G3BP2 NTF2L WT	SARS‐CoV‐2 N WT (1–25) MSDNGPQNQRNAPR**ITFG**GPSDSTG	10.9			
G3BP1 NTF2L WT	SARS‐CoV‐2 N WT (1–25) MSDNGPQNQRNAPR**ITFG**GPSDSTG	8.5			
G3BP1 NTF2L WT	SARS‐CoV‐2 N I15A (1–25) MSDNGPQNQRNAPR **ATFG**GPSDSTG	106			
G3BP1 NTF2L WT	SARS‐CoV‐2 N T16A (1–25) MSDNGPQNQRNAPR**IAFG**GPSDSTG	31.6			
G3BP1 NTF2L WT	SARS‐CoV‐2 N F17A (1–25) MSDNGPQNQRNAPR**ITAG**GPSDSTG	n.d.			
G3BP1 NTF2L WT	SARS‐CoV‐2 N F17N (1–25) MSDNGPQNQRNAPR**ITNG**GPSDSTG	n.d.			
G3BP1 NTF2L WT	SARS‐CoV‐2 N G18T (1–25) MSDNGPQNQRNAPR**ITFT** GPSDSTG	61.2			
G3BP1 NTF2L V11A	SARS‐CoV‐2 N WT (1–25) MSDNGPQNQRNAPR**ITFG**GPSDSTG	89.4			
G3BP1 NTF2L F15A	SARS‐CoV‐2 N WT (1–25) MSDNGPQNQRNAPR**ITFG**GPSDSTG	n.d.			
G3BP1 NTF2L Q18A	SARS‐CoV‐2 N WT (1–25) MSDNGPQNQRNAPR**ITFG**GPSDSTG	9.6			
G3BP1 NTF2L F33A	SARS‐CoV‐2 N WT (1–25) MSDNGPQNQRNAPR**ITFG**GPSDSTG	5.2			
G3BP1 NTF2L F124A	SARS‐CoV‐2 N WT (1–25) MSDNGPQNQRNAPR**ITFG**GPSDSTG	172			
G3BP1 FL WT	CAPRIN1 WT (full‐length) 370‐**YNFI**‐373	0.7	ITC	20 mM Tris pH 7.5, 200 mM NaCl	Song et al. 2022
G3BP1 FL WT	CAPRIN1 F372A (full‐length) 370‐**YNAI**‐373	n.d.			
G3BP1 NTF2L WT	CAPRIN1 WT (347–386)	1.3			
G3BP1 NTF2L WT	CAPRIN1 WT (342–380)	3.1			
G3BP1 NTF2L WT	CAPRIN1 WT (352–380)	9.4			
G3BP1 NTF2L WT	CAPRIN1 WT (353–393)	18			
G3BP1 NTF2L WT	USP10 WT (1‐40) 8‐**YIFGDF**‐13	10			
G3BP1 NTF2L WT	USP10 WT (1–25) MALHSPQ**YIFGDF**SPDEFNQFFVTP	0.11	SPR	20 mM Hepes pH 7.5, 150 mM NaCl, 5% Glycerol, 0.01% Triton X‐100, 5% DMSO	Yang et al. 2024
G3BP1 NTF2L F33W	USP10 WT (1–25) MALHSPQ**YIFGDF**SPDEFNQFFVTP	25.2			
G3BP1 NTF2L WT	SFV nsP3 WT (449–473) **LTFGDF**DEHEVDALASG**ITFGDF**DD	2.5			
G3BP1 NTF2L F33W	SFV nsP3 WT (449–473) **LTFGDF**DEHEVDALASG**ITFGDF**DD	n.d.			
G3BP1 NTF2L WT	CAPRIN1 WT (361–381) QDLMAQMQGP**YNFI**QDSMLDFE	13			
G3BP1 NTF2L F33W	CAPRIN1 WT (361–381) QDLMAQMQGP**YNFI**QDSMLDFE	n.d.			
G3BP1 NTF2L WT	SARS‐CoV‐2 N WT (1–25) MSDNGPQNQRNAPR**ITFG**GPSDSTG	1.14			
G3BP1 NTF2L F33W	SARS‐CoV‐2 N WT (1–25) MSDNGPQNQRNAPR**ITFG**GPSDSTG	1.92			
G3BP1 NTF2L WT	SFV nsP3 WT (449–473) **LTFGDF**DEHEVDALASG**ITFGDF**DD	0.2	SPR	20 mM Tris pH 7.4, 300 mM NaCl, 0.05% Tween‐20, 2% DMSO	Freibaum et al. 2024
G3BP1 NTF2L WT	Peptide mimetic G3Ia	0.54			
G3BP1 NTF2L WT	Peptide mimetic G3Ia′ (enantiomer)	75.5			
G3BP1 NTF2L WT	Peptide mimetic G3Ib	0.15			
G3BP1 NTF2L WT	Peptide mimetic G3Ib′ (enantiomer)	44.5			

In addition to G3BP1, a G3BP2 NTF2L domain homodimer was also co‐crystallized with a short peptide containing the SFV nsP3 ΦxFGDF_N_ motif, binding in the exact same fashion (Table 1), (Kristensen 2015), suggesting that G3BP2 could also form higher‐order oligomers with nsP3. Indeed, depletion of both G3BP1 and G3BP2 severely reduces the alphaviral RNA replication levels (Scholte et al. 2015). Other Old World alphavirus nsP3 proteins containing the ΦxFGDF were also shown to colocalize with G3BP proteins, as reported for instance for the Southeast Asian Getah virus (Qi et al. 2024). Interestingly, as shown for Mayaro virus, some Old World alphavirus nsP3 proteins contain a single canonical FGxF motif (Neyret et al. 2022). On the other hand, nsP3 proteins from Venezuelan and Western equine encephalitis New World alphaviruses, containing the Agenet‐like domain binding motif, colocalize with FMRP, another SG component (Kim et al. 2016; Nowee et al. 2021).

### 3.2 Bunyaviruses

Bunyaviruses are a large and diverse group of negative‐sense segmented RNA viruses. Depending on the family, they have developed different strategies to circumvent cellular translation shut‐off and the innate response. Orthobunyaviruses, like the La Crosse virus or the prototype Bunyamwera virus, were shown to trigger eIF2α phosphorylation in the infected mammalian cells (Hodges and Connor 2013; Streitenfeld et al. 2003), but little is known about SG assembly or disassembly during orthobunyaviral infection. However, transiently transfected Schmallenberg orthobunyavirus N protein was shown to localize in the arsenite‐triggered G3BP1‐positive foci (Xu et al. 2022).

On the other hand, Junín arenavirus N and GPC proteins were shown to prevent eIF2α phosphorylation and SG formation (Linero et al. 2011). Yet, proteomic analyses of the Junín and Lassa arenavirus nucleoprotein interactomes point to DDX3, G3BP1, AIFM1, and PABPC1 as potential SG‐related binding partners (King et al. 2017; Loureiro et al. 2018). Although not explored by alternative biochemical methods, it has been shown that Tacaribe and Junín arenavirus nucleoproteins sequester G3BP1 and a subset of ribosomal proteins and translation initiation factors into viral replication‐transcription complexes (Baird et al. 2012). Similarly, Tula hantavirus N protein co‐localizes with SG‐resident TIA1 protein, sequestering it to the filamentous viral factories built from structurally remodeled Golgi compartments, and possibly the redistributed SG components (Davies et al. 2020). In addition, infection with Puumala and Andes hantaviruses causes transient PKR‐dependent SG formation, limited to a fraction of cells, most probably because hantaviruses tend to prevent PKR‐ and PERK‐mediated stress signaling (Christ et al. 2020). Rift Valley fever phenuivirus prevents PKR‐dependent eIF2α phosphorylation (Ikegami et al. 2009) but induces mTOR pathway attenuation. The latter results in translational arrest via 5′‐TOP mRNA decay, and initially triggered SG accumulation is disrupted within a few hours post infection (Hopkins et al. 2015).

### 3.3 Coronaviruses

#### 3.3.1 Stress Granules in Coronaviral Infections and Determination of N–G3BP1 Interaction

Coronaviruses are single‐stranded, positive‐sense RNA viruses with large genomes of around 30 kb. They infect a variety of host cells across different species and evade host defense mechanisms in several ways, including the downregulation of SG formation and function. This is mainly observed in alpha‐coronaviruses like SARS‐CoV and SARS‐CoV‐2 (Li et al. 2022), MERS‐CoV (Nakagawa et al. 2018), and HCoV‐OC43 (Dolliver et al. 2022). Although some coronaviruses, like Porcine Epidemic Diarrhea Virus (PEDV) (Sun, Chen, et al. 2021; Zheng et al. 2024), and Mouse Hepatitis Virus (MHV) (Raaben et al. 2007), induce stress granules at early stages but they limit SG formation at later stages. The SARS‐CoV‐2 pandemic led to an unprecedented increase in basic research on coronavirus molecular biology, including the role of stress granules and SG‐associated factors during coronavirus infection. For instance, the proteomic peptide‐phage display approach (ProP‐PD), which screens for potential interactions between globular domains of the host factors and viral SLiMs, identified a coronaviral strain‐specific interaction between the N‐terminus of the SARS‐CoV‐2 and SARS‐CoV N nucleocapsid protein and the G3BP1/2 NTF2 domain (Kruse et al. 2021), which was also suggested in large screens of the SARS‐CoV‐2 interactome (Gordon et al. 2020; Li et al. 2021; Nabeel‐Shah et al. 2022). Perrin‐Cocon et al. (2020) and Baggen et al. (2021) provided an extensive overview of host‐virus protein–protein interactions, determined for major coronaviruses. G3BP1 was previously identified as well among interactors of the infectious bronchitis coronavirus (IBV) N protein (Emmott et al. 2013). It was proposed that SARS‐CoV‐2 and MERS‐CoV N proteins impair SG formation by inhibiting PKR autophosphorylation and activation (Zheng et al. 2021). The SARS‐CoV‐2 N protein was demonstrated to undergo an RNA‐ and N‐terminus‐dependent LLPS with G3BPs, leading initially to the N protein colocalization within SGs and later to their disassembly (Lu et al. 2021; Luo et al. 2021; Wang et al. 2021; Yang et al. 2024). At the same time, G3BPs were postulated to drive the condensation of the SARS‐CoV‐2 RNAs, dysregulating viral replication and antagonizing subsequent infection events (Burke et al. 2024).

#### 3.3.2 Details of the SARS‐CoV‐2 N–G3BP1 Interaction

Coronaviral N proteins are composed of an N‐terminal RNA‐binding domain (RBD) and a C‐terminal homodimerization domain (HDD), flanked by three IDRs (Figure 2F). All‐atom simulations and FRET assays, performed on the SARS‐CoV‐2 N protein, revealed that all three IDRs are expanded and dynamic, while both globular domains interact minimally, contributing to a flexible and multivalent nature of the protein (Cubuk et al. 2021). The first IDR of the SARS‐CoV‐2 N protein contains a USP10‐like ΦxFG SLiM (Figure 2G) (Kruse et al. 2021), which binds within the G3BP1 NTF2L domain with high affinity (Table 2) and within the four main residues in the same fashion as USP10 or SFV nsP3 SLiM (Biswal et al. 2022), (Table 1), (Figure 2H). SARS‐CoV‐2 N protein residues I15 and F17 lie within the G3BP1 NTF2L hydrophobic grove, with the F17 residue accommodated in the NTF2L F15/F33 pocket. In addition, the peptide turn, provided by the G18 and G19 flexibility and stabilized by NTF2L K123, positions N protein P20 towards E117 and Y125 from the NTF2L β‐sheet. Mutations in any of the four ΦxFG SLiM residues affect the binding affinity, with the F17 mutation completely disrupting it (Biswal et al. 2022).

The middle SARS‐CoV‐2 N protein IDR (residues 176–206) is SR‐rich and its phosphorylation by SRPK1 kinase regulates both formation of the viral RNP higher‐order complexes and localization of the N proteins in SGs. Positive charges of the SR dipeptide arginines contribute to the N protein LLPS dynamics and stability, while phosphorylation of serines counter‐balances arginines, inhibiting N localization in SGs (Lu et al. 2021; Peng et al. 2008). On the other hand, methylation of arginine R95 by cellular PRMT1 was shown to prevent N‐mediated SG disassembly (Cai et al. 2021). Apart from SARS‐CoV‐2 N protein IDRs, both N‐terminal RBD and C‐terminal HDD domains are also important for the N protein phase separation. Deletion of the C‐terminal HDD together with C‐terminal IDR increases N protein diffusion from LLPS droplets in vitro, suggesting that while the N‐terminal ΦxFG SLiM triggers the N protein LLPS with G3BP1, the C‐terminus stabilizes it presumably due to homodimerization (Luo et al. 2021). It might well be that G3BP1 and SARS‐CoV‐2 N homodimerizations could potentially lead to the formation of higher‐order oligomers. Additionally, the N protein RBD domain is also involved in mediating SG disassembly, as its removal deprives the N protein of its ability to prevent RNA‐dependent G3BP1 droplet formation (Huang et al. 2021).

In the later stages of coronaviral infection, high levels of N may outcompete other ΦxFG‐containing SG interactors of G3BP1, further facilitating the SG disruption (Kruse et al. 2021; Nabeel‐Shah et al. 2022). At the same time, RNA‐mediated SARS‐CoV‐2 N protein phase separation enables viral RNA genome condensation and compaction during virion assembly (Cubuk et al. 2021), and facilitates the recruitment of the viral RNA‐dependent RNA polymerase complexes (Savastano et al. 2020). Interestingly, proteomic analysis of the SARS‐CoV‐2 virions revealed that G3BP1 and G3BP2 are significantly enriched among embedded cellular proteins, proving that the N–G3BP1 interaction is most likely maintained inside progeny virions. In fact, G3BP proteins might be positively involved in the viral RNA packaging and virion assembly, since the simultaneous knock‐down of both G3BP1 and G3BP2 led to decreased production of infectious virions (Murigneux et al. 2024). This dual function of the coronaviral N–G3BP interaction opens the possibility for a design of peptide‐based inhibitors, which would not only restore SG formation but also prevent SARS‐CoV‐2 virion assembly (Kruse et al. 2021).

#### 3.3.3 Other Coronaviral Proteins Contacting SG Components

The SARS‐CoV‐2 nsp3 protein was also assigned as a potential SG interactor and disruptor. Based on immunopurification assays and AlphaFold modeling, a 20‐aa fragment from the hypervariable region of this large and multi‐functional viral protein was shown to interact with the fragile X mental retardation proteins (FMRPs: FMR1, FXR1‐2). Binding of the SARS‐CoV‐2 nsp3 protein fragment to the two FMRPs KH domains disrupts the FMRPs contact with UBAP2L, thus dysregulating the SG network (Garvanska et al. 2024). Biotinylation proximity assay revealed as well that nsp1protein of both SARS‐CoV and SARS‐CoV‐2 associates with G3BPs and other SG proteins (Gerassimovich et al. 2021). This might be related to sequestration of the nsp1‐inhibited 40S ribosomal subunits (Schubert et al. 2020; Thoms et al. 2020). In addition, SARS‐CoV‐2 and HCoV‐OC43 nsp1 proteins were shown to inhibit arsenite‐induced eIF2α phosphorylation, while the former one additionally promotes nuclear accumulation of another SG‐nucleating protein TIAR (Dolliver et al. 2022).

Coronaviral endonuclease nsp15 interferes as well in the antiviral SG formation, as demonstrated with IBV, MHV‐A59, and PEDV coronaviruses. Supposedly, this is achieved by a cumulative effect of cleavage of both the host mRNAs and the 5′‐poly(U) sequences from the negative‐sense viral genome intermediate. The latter activity of nsp15 prevents the accumulation of the viral dsRNA, thus antagonizing the activation of the pattern recognition receptors like MDA5, OAS, or PKR (Gao et al. 2021; Hackbart et al. 2020). In an alternative approach, MERS‐CoV accessory protein 4a shields viral dsRNA, preventing its recognition by cellular receptors, resulting among other things in the inhibition of PKR‐mediated eIF2α phosphorylation (Nakagawa et al. 2018; Rabouw et al. 2016). The SARS‐CoV‐2 nsp5 cysteine protease was also reported to suppress SG formation and disrupt the RIG‐I–MAVS complex, attenuating the RIG‐I‐mediated antiviral signaling (Zheng et al. 2022). Nsp5 was shown to cleave off 10 amino‐terminal RIG‐I residues, preventing it from activating MAVS (Liu et al. 2021). Bioinformatic screen proposed other possible nsp5 targets among cellular proteins (Scott et al. 2022).

#### 3.3.4 Coronaviral Genome Association With TIA1 Protein

Apart from host‐coronavirus protein–protein interactions, it has been proposed that TIA1, another stress granule factor, interacts with the 5′ UTR of the SARS‐CoV‐2 genome. Coronavirus genomic UTRs and other positive‐sense virus genomes are highly structured and harbor several stem‐loops, serving as RNA–RNA and RNA–protein cis regulatory elements. 42 functionally related host cell proteins were predicted to bind to the SARS‐CoV‐2 RNA genome, with TIA1 protein among them (Sun, Li, et al. 2021). TIA1 is a ubiquitous RNA‐binding protein composed of three RRM domains, broadly involved in gene expression regulation. EMSA assays and MD simulations revealed that the SL3 element of the SARS‐CoV‐2 genome can interact tightly with the TIA1 RRM domains 2 and 3, possibly sequestering the protein to the viral replication sites (Zhang et al. 2023).

### 3.4 Flaviviruses

#### 3.4.1 Flaviviral RNAs and SG Components

Similarly to coronaviruses, UTRs of the positive‐sense flaviviral genomes are highly structured and functionally involved in regulating replication and translation. If they happen to undergo G3BP1‐mediated condensation, they become translationally incompatible. In order to defend against that scenario, flaviviruses like West Nile virus (WNV) or Zika virus (ZIKV) hijack the RNA decondensing activity of eIF4A or counteract the RNA‐binding capacity of G3BP1 (Burke et al. 2024). The latter can be achieved with highly structured, subgenomic flaviviral 3′ UTR RNAs (sfRNAs) – products of the incomplete, XRN1‐mediated degradation of the flaviviral genome (Chapman et al. 2014; Zhang, Zhang, et al. 2019). Flaviviral sfRNAs can sponge SG proteins, either preventing G3BP1 from condensing the actual genomic RNAs (Burke et al. 2024) or counteracting SG formation or the IFN response stimulation (Bidet et al. 2014). Hepatitis C virus (HCV) and Dengue virus (DENV) 3′ UTR sfRNAs were shown to interact with G3BPs, PABPC1, CAPRIN1, and USP10 (Bidet et al. 2014; Tingting et al. 2006; Ward et al. 2011). At the same time, G3BP1 RG‐rich IDR3 was shown to associate with the RIG‐I helicase, assisting it in binding and recognizing HCV dsRNA replication intermediates, essentially acting as a co‐sensor of the viral dsRNA (Kim et al. 2019).

SG proteins can also be recruited or hijacked by RNA elements of the flaviviral genome to promote viral translation or replication. Staufen1 was shown to interact both with the HCV 5′ UTR IRES, promoting translation, as well as with the 3′ UTR stem‐loops and NS5B viral polymerase, assisting in genome replication (Dixit et al. 2016). DEAD‐box RNA helicase DDX3 is involved in the translation initiation complex assembly on the HCV IRES (Geissler et al. 2012), but was also shown to bind 3′ UTR, allowing for IKK‐α activation, which triggers lipogenesis and therefore facilitates viral assembly (Li et al. 2013; Pène et al. 2015). HuR protein was shown to bind the HCV genomic and anti‐genomic 3′ UTRs and contribute to HCV replication (Korf et al. 2005; Spångberg et al. 2000). It was also reported that TIA1 and its related protein TIAR may interact with structured elements of the West Nile virus, Dengue virus, and tick‐borne encephalitis virus genomic 5′ UTR (Albornoz et al. 2014; Emara and Brinton 2007) or anti‐genome 3′ promoter (Li et al. 2002). Interaction with these stem‐loops recruits TIA1/TIAR as potential pro‐replication factors to the viral replication sites, causing at the same time the depletion of the stress granules. This is in line with the TIAR knockout cells infected with West Nile virus yielding lower viral titers than regular cells (Li et al. 2002).

#### 3.4.2 SG Dynamics and SG Proteins Repurposing During Flaviviral Infection

Flaviviruses were shown to use various strategies to counteract SG formation, either by regulating the level of the early viral RNA synthesis (Courtney et al. 2012), stimulating antioxidant gene expression (Basu et al. 2017), modulating eIF2α dephosphorylation (Amorim et al. 2017; Wu et al. 2024), or by suppressing downstream PKR signaling without necessarily affecting the eIF2α(P)‐mediated host translation shut‐off (Arakawa et al. 2022). While some flaviviruses, like WNV or ZIKV, prevent or limit SG formation (Bonenfant et al. 2019; Courtney et al. 2012), others, like DENV, induce a delayed SG formation (Xia et al. 2015), or like HCV, trigger temporary SG formation, followed by their dissociation in the later infection phase (Ariumi et al. 2011). On the other hand, as demonstrated by detailed time‐course analysis, cells persistently infected with HCV undergo a dynamic oscillation of the SG assembly and disassembly, correlated with phases of stalled and active translation and mediated by PKR‐dependent phosphorylation and PPI‐dependent dephosphorylation of eIF2α. Such fluctuations of the stress response prevent, on one hand, long‐lasting translation repression, at the same time allowing the virus to establish a persistent infection (Klein et al. 2022; Ruggieri et al. 2012).

SG disassembly during flaviviral infection correlates with redistribution of SG‐associated proteins like G3BP1, ATX2, and PABPC1 to the HCV membrane‐associated replication organelles (Ariumi et al. 2011; Bonenfant et al. 2019; Garaigorta et al. 2012; Pager et al. 2013). G3BP1 was found, for instance, to be associated with the HCV NS5B polymerase within these viral compartments, where it might be co‐opted as a functional replication complex component (Yi et al. 2006, 2011). The capsid protein of the Japanese encephalitis virus or ZIKV, whose main role is to anchor the viral genome inside the virion, was shown by IP–MS and by in vitro pull‐down to interact with CAPRIN1, preventing SG formation (Hou et al. 2017; Katoh et al. 2013). Mutagenesis revealed that the capsid protein positively charged residues K97 and R98 are crucial for that interaction (Katoh et al. 2013). DDX3 helicase, possibly due to its connection with the HCV capsid protein, was shown to be required for HCV genome replication (Ariumi et al. 2007). Conversely, the N‐terminal domain of the DENV capsid protein has been reported to interact with the N‐terminal domain of the DDX3 helicase, leading to down‐regulation of DENV infection (Kumar et al. 2017). DDX3 and YB‐1 were also shown to interact with the pivotal viral phosphoprotein NS5A, supposedly participating in connecting the 5′ and 3′ ends of the flaviviral genome and impacting various steps of the HCV life cycle (Wang et al. 2015). Finally, ZIKV non‐structural proteins were also postulated to participate in preventing SG formation (Machmouchi et al. 2024).

### 3.5 Influenza Viruses

Influenza viruses trigger the shutoff of cellular translation by combined actions of non‐structural protein 1 (NS1) and polymerase acidic X protein (PA‐X). While NS1 inhibits host pre‐mRNA maturation and export from the nucleus, PA‐X endonucleolytically cleaves host cytoplasmic mRNAs (Bougon et al. 2024; Khaperskyy et al. 2016). Nuclear retention and degradation of the poly(A) RNAs during influenza virus infection lead consequently to nuclear accumulation of PABPC1 (Khaperskyy et al. 2014). Importantly, influenza virus mRNAs are primed by the viral polymerase with host‐derived, 5′‐capped oligonucleotides and contain poly(A) tail, which makes them vulnerable to the overall shutoff of the canonical, 5′‐cap‐dependent translation. While this manifests at early stages of infection, at later stages viral mRNAs become resistant to the translation arrest.

In line with that, influenza viruses tend to block stress‐induced SG formation by concerted action of three viral proteins: NS1, PA‐X, and N nucleoprotein (Khaperskyy et al. 2012). NS1 binds viral dsRNA, shielding it from recognition by PKR, and thus preventing eIF2α phosphorylation. On the other hand, the IP–MS screen revealed that NS1 associates with the PB and SG factor RAP55 (Mok et al. 2012). Ectopically expressed N protein was shown to prevent arsenite‐triggered SG formation. Its oligomerization capacity seemed to be important in this regard, as mutagenesis of residues involved in intermolecular N–N contacts abolished that effect (Khaperskyy et al. 2014). PA‐X, expressed at the later stage of infection, prevents SG formation indirectly by excessive degradation of the host cytoplasmic mRNA. IP interactome analysis suggested that PA‐X interacts with the CFIm complex, bridging splicing and polyadenylation during RNA processing, and is therefore set for degradation of the intron‐containing host transcripts (Gaucherand et al. 2019). DDX3 RNA helicase was also shown to act against influenza virus infection, as its knockdown resulted in impaired SG formation and increased virus titers (Raman et al. 2016).

### 3.6 Mononegavirales

*Mononegavirales* order groups negative‐sense RNA viruses with a single, long RNA genome and contains several human pathogens like Ebola virus (EBOV), human respiratory syncytial virus (RSV), measles virus, mumps virus, Nipah virus, or rabies virus. RSV was shown to induce SG formation in epithelial cells during the course of infection. RSV‐induced SGs are distinct from viral inclusion bodies and appear at the transition from viral genome transcription to replication. They were postulated to facilitate the RSV genome replication taking place within viral inclusion bodies (Lindquist et al. 2010). Measles virus was also shown to replicate within cytoplasmic inclusion bodies or membrane‐less viral factories containing condensed viral replication machinery and being unrelated to SGs or other cytoplasmic foci (Zhou et al. 2019). Measles virus infection induces, though, PKR‐dependent SG formation (Okonski and Samuel 2013). Apart from RSV, parainfluenza virus 5 and Sendai virus are also known to induce stress granules, although at relatively late time points post infection (Carlos et al. 2009; Iseni et al. 2002). In the latter case, trailer RNA, a short, abortive viral transcript, binds TIAR protein, potentially impacting the cellular antiviral response (Iseni et al. 2002). Supposedly, the formation of the viral inclusion bodies prevents or limits SG formation by shielding newly produced viral RNA (Hu et al. 2018). Similarly to other *Mononegavirales* viruses, vesicular stomatitis virus (VSV) also induces the formation of cytoplasmic, SG‐like viral replication foci, which contain some, like TIA1 and TIAR, but not all, classical SG‐associated proteins (Dinh et al. 2013). Detailed co‐immunostaining analysis of cells infected with rabies virus revealed that SGs induced during infection can localize in very close proximity to the viral replication foci, yet remain distinct (Nikolic et al. 2016). Ebola virus, on the other hand, was shown not to induce SG formation, with some SG proteins, like in previous examples, being sequestered into viral replication bodies where they colocalize with viral RNA (Nelson et al. 2016). Ebola virus protein VP35 was proposed to be directly involved in preventing SG formation (Le Sage et al. 2017; Nelson et al. 2016). A recent study reported that while bovine parainfluenza virus infection causes eIF2α phosphorylation, SGs do not form, as in Ebola's case, most likely due to downregulation of G3BP1 expression (Liu et al. 2024).

### 3.7 Picornaviruses

#### 3.7.1 G3BP1 Binds IRES and Negatively Regulates Picornaviral Translation

Picornaviruses are non‐enveloped, positive‐sense RNA viruses, relying on cap‐independent mechanisms to initiate translation. 5′ UTRs of their genome contain internal ribosome entry site (IRES) – defined three‐dimensional RNA structures, directly recruiting cellular RNA‐binding proteins and translation initiation factors, including the 40S small subunit of the ribosome (Martínez‐Salas et al. 2015). During picornaviral infection, cellular IRES trans‐acting factors (ITAFs) undergo various changes like redistribution between cellular compartments, adjustment of the phosphorylation and other PTM levels, or cleavage by picornaviral proteases. Stress granule factor G3BP1 is among cellular proteins binding to picornaviral IRES. It was shown to interact directly with the foot‐and‐mouth disease virus (FMDV) IRES, negatively regulating viral translation (Galan et al. 2017). Gel‐shift assays revealed that recombinant G3BP1 can interact with three distinct regions of the FMDV IRES, two structured elements and a single‐stranded region at the 3′ end of the IRES. The latter observation could suggest that the C‐terminal RRM domain of G3BP1 participates in binding the IRES. That could also explain why the C‐terminal product of the G3BP1 cleavage by the FMDV 3C protease, containing the RRM domain followed by the IDR3, inhibits picornaviral IRES‐dependent translation more strongly than the N‐terminal fragment, and similarly to the full‐length G3BP1 (Galan et al. 2017).

#### 3.7.2 G3BP1 Is Cleaved by Picornaviral Proteases

Cleavage of G3BP1 by the 3C protease between residues Q325 and G326 might be a general picornaviral strategy to mitigate G3BP1‐driven IRES suppression, as it has been reported not only for FMDV (Galan et al. 2017; Ye et al. 2018), but also for poliovirus (White et al. 2007), EMCV (Ng et al. 2013), coxsackievirus CVB3 (Fung et al. 2013), and Seneca Valley picornavirus (Wen et al. 2020). Additionally, it was proposed that the FMDV papain‐like L protease participates as well in G3BP1 cleavage (Visser et al. 2019). Additionally, cleavage of G3BP1 disables SG formation. As shown for poliovirus, while SGs are initially induced during the early phase of picornaviral infection, they disperse after several hours (White et al. 2007), presumably because separated N‐ and C‐terminal parts of G3BP1 lose the ability to form and maintain the SG protein–RNA network. Introduction of a cleavage‐resistant G3BP1 restores SG formation and affects poliovirus replication (White et al. 2007). Similarly, overexpression of G3BP1 reduces CVB3 viral protein expression, transcripts, and viral titers (Fung et al. 2013), altogether indicating that SGs negatively regulate picornaviral infection. Apart from G3BP1, picornaviruses target proteolytically other host factors involved in cellular gene expression, like eIF4GI, eIF4GII, PABPC1 (Lloyd 2006), factors involved in Pol I, Pol II, and Pol III transcription (Weidman et al. 2003), or nuclear pore proteins (Gustin and Sarnow 2001), altogether interfering with the cell's ability to activate the antiviral response.

#### 3.7.3 Caliciviruses Redistribute G3BP1 Into Viral Replication Centers

*Caliciviridae* family belongs to picornaviruses and accounts for the human norovirus (HuNoV) and the closely related murine norovirus (MNV). The latter, broadly used as the calicivirus model, was shown to activate ISR and shut off the host cell translation, although independently from the PKR‐mediated eIF2α phosphorylation and without stimulating the SG formation (Brocard et al. 2020; Fritzlar et al. 2019). Lack of SG assembly during noroviral infection can possibly be a consequence of the G3BP1 interactome remodeling and, related to that, its redistribution towards viral replication centers (Brocard et al. 2020; Hosmillo et al. 2019). Translation of the noroviral proteins is mediated by the genome‐linked VPg protein, recruiting eIF3 translation initiation factor (Daughenbaugh et al. 2003, 2006), either directly or indirectly, via its interaction with the eIF4F components (Goodfellow et al. 2005; Leen et al. 2016). Once redistributed to the viral replication centers, G3BP1 contributes to promoting the VPg‐dependent translation (Hosmillo et al. 2019). In this regard, a CRISPR screen demonstrated that G3BP1 is critical for facilitating MNV infection and replication (Orchard et al. 2016). G3BP1 RRM and RG‐rich IDR3 region possibly mediate its protein–protein and/or protein–RNA connections within noroviral replication sites (Fritzlar et al. 2019; Hosmillo et al. 2019), although the exact mechanism remains unclear.

Unlike MNV, the Feline calicivirus NS6 protease was shown to cleave G3BPs, reducing the SG formation in that way (Humoud et al. 2016).

### 3.8 Reoviruses

Infection with mammalian orthoreovirus (MRV) (family *Spinareoviridae*) induces phosphorylation of translation initiation factor eIF2α, which promotes SG formation at early times of infection and shuts down cellular translation. As orthoreoviral infection progresses, SG formation gets interfered with and remains prevented, regardless of the eIF2α(P) (Qin et al. 2009). Disruption of SGs during MRV is necessary to enable orthoreoviral translation despite eIF2α(P) (Qin et al. 2011) and is achieved by re‐localization of the SG‐associated proteins to the peripheries of the viral factories (VFs), virus‐encoded cytoplasmic compartments, where viral replication, transcription, and translation occur (Desmet et al. 2014). Major SG factor G3BP1 gets recruited to the orthoreoviral VFs through its association with the MRV protein σNS, anchored to the VF‐forming protein μNS (Choudhury et al. 2017). Other SG‐associated proteins like CAPRIN1, TIAR, and TIA1 are re‐distributed as well to the VF peripheries, potentially due to their connection with G3BP1. Domain deletion analysis revealed that both G3BP1 RRM and RG‐rich IDR3 participate in association with MRV σNS and are consequently necessary for efficient recruitment into orthoreoviral VFs (Choudhury et al. 2017).

Infection with rotaviruses (family *Sedoreoviridae*) leads as well to the eIF2α phosphorylation, dependent on three viral proteins, VP2, NSP2, and NSP5. G3BP1 appears to negatively regulate rotavirus infection. Therefore, similarly to orthoreoviruses, rotaviruses prevent the formation of the G3BP1‐positive SGs, causing a re‐distribution of SG‐associated proteins (Montero et al. 2008), or even a sequestration of atypical, remodeled SGs, devoid of G3BP1, into rotaviral viroplasms (Dhillon et al. 2018; Dhillon and Rao 2018).

## 4 Concluding Remarks

While many viruses repurpose or antagonize SG formation, molecular details of these mechanisms remain vague, as in many cases, only the IF co‐localizations or IP‐based interactions are available. Although these approaches offer valuable insights, they often do not provide the mechanistic clarity needed to fully understand the nature and functional implications of the interactions between viral factors and SG components. Only in a handful of cases discussed in this review were detailed structural and biochemical aspects of such interactions reported. Broadening our understanding in this area is crucial, as it will not only deepen our knowledge of the viral strategies for seizing control of infected cells and evading their immune response but will also pave the way for developing targeted antiviral drugs and targeted therapies, as exemplified by Freibaum et al. (2024).

## Author Contributions

**Moh Egy Rahman Firdaus:** conceptualization (supporting), writing – original draft (supporting), writing – review and editing (supporting). **Eliana Dukhno:** writing – original draft (supporting), writing – review and editing (supporting). **Rupali Kapoor:** writing – original draft (supporting), writing – review and editing (supporting). **Piotr Gerlach:** conceptualization (lead), funding acquisition (lead), visualization (lead), writing – original draft (lead), writing – review and editing (lead).

## Conflicts of Interest

The authors declare no conflicts of interest.

## Related WIREs Articles

Regulation of stress granules and P‐bodies during RNA virus infection

Stress‐induced mRNP granules: Form and function of processing bodies and stress granules

Changing faces of stress: Impact of heat and arsenite treatment on the composition of stress granules

A little less aggregation a little more replication: Viral manipulation of stress granules

## Data Availability

Data sharing are not applicable to this article as no new data were created or analyzed in this study.
